# Intact lymph node homing and CD8^+^ T-cell priming abilities of Sprouty2-deficient dendritic cells

**DOI:** 10.3389/fimmu.2026.1761675

**Published:** 2026-03-18

**Authors:** Nadine Anslinger, Guerric P. B. Samson, Vladimir Purvanov, Michael Basler, Daniel F. Legler

**Affiliations:** 1 Institute of Cell Biology and Immunology Thurgau (BITG) at the University of Konstanz, Kreuzlingen, Switzerland; 2 Graduate School for Cellular and Biomedical Sciences (GCB), University of Bern, Bern, Switzerland; 3 Department of Biology, University of Konstanz, Konstanz, Germany; 4 Theodor Kocher Institute, University of Bern, Bern, Switzerland

**Keywords:** CD8^+^ T-cell priming, chemokine receptor CCR7, chemotaxis, dendritic cells, lymph node homing, Sprouty2

## Abstract

Sprouty2 (Spry2) acts as a modulator of the MAPK-ERK signaling pathway by exerting both positive and negative regulation in a highly context- and cell type-specific manner. While its role in controlling the migration of non-immune cells in growth factor-dependent contexts is well established and continuously expanding, its function as a modulator of immune cell responses has only recently begun to emerge. Spry2 appears to critically and differentially influence B- and T-cell responses, consistent with its cell type-specific nature. However, its role in dendritic cells (DCs) remains unexplored. DCs serve as the cellular link between innate and adaptive immunity, and hence, DCs rely on their ability to navigate through different tissues and migrate to distinct target locations to initiate and coordinate effective immune responses. In the present study, we show that Spry2 expression is regulated during bone marrow-derived (BM)DC maturation and established a CD11c-specific Spry2 knockout mouse model to analyze the *in vitro* and *in vivo* immune functions of Spry2-deficient DCs. Unexpectedly, we found that its complete absence does not alter essential DC immune functions. Spry2-deficient DCs display intact DC differentiation *in vitro* and *in vivo*, efficient CCR7-driven migration and effective lymph node homing *in vivo*. Furthermore, we demonstrate that Spry2-deficient DCs retain an unaltered ability to stimulate CD8^+^ T-cell activation and proliferation, ultimately resulting in normal CD8^+^ T-cell effector differentiation during acute viral infection. Collectively, our findings shed light on the function of Spry2 in DCs, thereby extending and reinforcing current knowledge of its diverse immunomodulatory functions.

## 1 Introduction

The Sprouty (Spry) protein was originally identified in *Drosophila* as an antagonist of fibroblast growth factor signaling during tracheal branching (1). Since then, four mammalian homologues, Spry1–4, have been identified based on sequence similarity (1–4). Extensive research has characterized Spry proteins as key regulators of the MAPK-ERK signaling pathway. They interact with an increasing number of binding partners, thereby modulating ERK activity either positively or negatively, and can also mediate crosstalk with other signaling pathways. As a result, their modes and sites of action are highly context- and cell type-dependent. In this way, Spry proteins influence key cellular processes, including cell proliferation, differentiation, motility and survival, and play a vital role during embryogenesis and in adult organ physiology (5–8). Hence, it is not surprising that Spry dysregulation has been linked to cancer development and metastasis formation, either as tumor suppressors or as oncogenes (7–9). As the most evolutionary conserved isoform, Spry2 seems to be ubiquitously expressed in embryonic and adult tissues (3, 7, 10). Notably, Spry2 has been implicated in controlling cell migration of non-immune cells, mostly in context of growth factor signaling and wound healing assays (11–23). Interestingly, overexpression of mouse Spry2 in the osteosarcoma cell line LM8 suppresses tumor metastasis formation and inhibits chemotactic cell migration in response to the chemokines CCL19 and CCL21 (24), the cognate ligands of the chemokine receptor CCR7. While expression of CCR7 by cancer cells has been shown to facilitate migration and metastasis (25), its main physiological function is to coordinate immune cell homing to lymphoid organs (26). In addition, emerging evidence has identified Spry2 as a versatile modulator of immune cell functions, aligning with its context- and cell type-specific function. In B cells, Spry2 is upregulated following B-cell receptor (BCR) stimulation and acts as a negative regulator of the MAPK-ERK pathway, thereby attenuating BCR signaling and reducing cell survival and proliferation (27, 28). Similarly, Spry2 is induced in CD4^+^ and CD8^+^ T cells upon T-cell receptor (TCR) engagement, however, its functional role seems to be T-cell subset specific. For human CD8^+^ T cells, Chiu and colleagues reported that elevated Spry2 levels during chronic viral infection decrease their ability to simultaneously secrete multiple cytokines, thereby compromising T-cell polyfunctionality (29). In addition, Shehata et al. showed that a conjoint loss of mouse Spry1 and 2 increases effector CD8^+^ T-cell survival during the contraction phase, resulting in a higher number of protective memory cells and improved recall responses across different infection models (30). While the lack of Spry1/2 in CD8^+^ T cells seems to positively influence memory formation, absence of Spry2 in CD4^+^ T cells critically impedes CD4^+^ T-cell function. Spry2-deficient mouse CD4^+^ T cells showed defective TCR signaling, reduced proliferation as well as impaired T helper-cell differentiation and cytokine production. Mechanistically, Spry2 was identified as a positive regulator of ERK signaling in CD4^+^ T cells by modulating LCK activity (31). While Spry2 has been shown to exert important regulatory functions in B and T cells, its role in other immune cells, such as dendritic cells (DCs), remains largely unexplored. As highly specialized antigen-presenting cells, DCs play a central role in bridging innate and adaptive immunity. In their immature state, they act as sentinels of the immune system, continuously scanning peripheral tissues and capturing antigens for processing (32). Upon pathogen encounter, they undergo a maturation process characterized by the upregulation of co-stimulatory molecules and the chemokine receptor CCR7 (33–37). Once mature, antigen-bearing DCs migrate in a CCR7-dependent manner to draining lymph nodes (dLNs), where they present antigens to cognate T cells and initiate an adaptive immune response (34, 38, 39). Effective navigation through different tissue environments and targeted migration to specific locations are therefore essential for DC functions. Notably, interference with ERK signaling has been shown to alter DC responses, including survival (40, 41), maturation (42–45), CCR7-driven migration (44–49) and T-cell priming (50). For example, recent work has shown that sustained ERK activity inhibits CCR7-mediated DC migration to dLNs, contributing to trapping of DCs in tissue lesions during the pathological context of Langerhans cell histiocytosis. Notably, DC migration could be restored in a therapeutic approach by using a nanoparticle-based MAPK inhibitor delivery system targeting DCs via phagocytosis (49). Furthermore, ERK1-deficient DCs were shown to exhibit enhanced migratory capacities and potentiate DC vaccination in the context of high-grade gliomas, thereby identifying ERK1 as a potential therapeutic target (48). In conclusion, investigating potential ERK regulators in DCs and their impact on DC biology is highly relevant, especially considering the therapeutic potential of DCs in immunotherapy (51). In this study, we assessed the role of the known ERK modulator Spry2 in DCs by analyzing the *in vitro* and *in vivo* functions of Spry2-proficient and -deficient DCs in context of CCR7-driven migration, LN homing and CD8^+^ T-cell priming. Although Spry2 expression is regulated during bone marrow-derived (BM)DC maturation, we observed normal DC differentiation both *in vitro* and *in vivo* as well as efficient LN homing and CD8^+^ T-cell priming in the absence of Spry2. Hence, we found no evidence that Spry2 deficiency positively or negatively influences DC functions, which is in contrast to its reported role in T and B cells. Collectively, our data provide insight into the role of Spry2 in DCs and corroborate the context-dependent nature of Spry2 function across immune cell types.

## 2 Materials and methods

### 2.1 Mice

Mice were housed and bred in a specific pathogen-free facility at the University of Konstanz. C57BL/6J (H-2^b^) mice were originally purchased from Charles River, Germany. OT-I (C57BL/6-Tg (TcraTcrb)1100Mjb) were originally provided by the Swiss Immunological Mutant Mouse Repository. *CD11c-Cre* (C57BL/6-Tg(Itgax-cre)/Rj) (52) mice were originally obtained from Christian Münz, University of Zürich. *Spry2^fl^* mice (53) (STOCK *Spry2^tm1Mrt^*/Mmnc, RRID: MMRRC_011469-UNC) were obtained from the Mutant Mouse Resource and Research Center (MMRRC) at University of North Carolina at Chapel Hill, an NIH-funded strain repository, and was donated to the MMRRC by Gail R. Martin, University of California, San Francisco. *Spry2^fl^* mice were intercrossed and further crossed to *CD11c-Cre* mice to generate mice with a CD11c-specific deletion of Spry2. Sex- and age-matched *Spry2^+/+^CD11c-Cre^+^* (Spry2^+/+^), *Spry2^+/fl^CD11c-Cre^+^* (Spry2^+/-^) and *Spry2^fl/fl^CD11c-Cre^+^* (Spry2^-/-^) mice were used throughout the study. Mice aged 6–12 weeks were used for animal experiments and mice aged 6–20 weeks were used for organ collection. Animal experiments and organ collection from mice were approved by the Review Board of the Regierungspräsidium Freiburg, Baden-Württemberg, Germany (T-21/03TFA, T-24/02TFA, G-21/089 and G-23/048).

### 2.2 Tissue lysis and genomic PCR for genotyping

Tissue from ear punching was used for genotyping. The KAPA HotStart Mouse Genotyping Kit (Roche, Basel, Switzerland) was used for extraction of genomic DNA and PCR amplification according to the manufacturer’s guidelines. Primers specific for the Spry2 loxP sites (5´-AATAGGGATTGTTGCTCCG-3´ and 5´-GCATGGGCTATTCACAAC-3´ (53)) and the CD11c-Cre transgene (5´-GCGGTCTGGCAGTAAAAACTATC-3´and 5´-GTGAAACAGCATTGCTGTCACTT-3´) were used. The expected PCR product sizes were 330 bp for the WT *Spry2* allele, 375 bp for the floxed *Spry2* allele and 100 bp for the *Cre* transgene. PCR products were separated by gel electrophoresis on a 2% agarose gel and visualized using SafeView™ Classic DNA stain (Applied Biological Materials Inc., Richmond, BC, Canada) and a gel imaging system (Bio-Rad Laboratories, Hercules, CA, USA).

### 2.3 Generation of GM-CSF bone marrow-derived (BM)DCs

BMDCs were generated as described previously (54). Bone marrow was isolated from femur and tibia of *Spry2^+/+^CD11c-Cre^+^* (Spry2^+/+^), *Spry^+/fl^CD11c-Cre^+^* (Spry2^+/-^) and *Spry2^fl/fl^CD11c-Cre^+^* (Spry2^-/-^) mice. Cell suspension was depleted of red blood cells using red blood cell (RBC) lysing buffer (BioLegend, San Diego, CA, USA). Subsequently, 3 x 10^6^ cells were seeded in 94 mm petri dishes (Greiner Bio-One, Kremsmünster, Austria) in 10 mL R10 media supplemented with 20 ng/mL mouse granulocyte-macrophage colony-stimulating factor (GM-CSF; PeproTech, ThermoFisher Scientific, Waltham, MA, USA; #315-03-100UG). R10 media was composed of Roswell Park Memorial Institute (RPMI) 1640 medium (PAN-Biotech, Aidenbach, Germany) supplemented with 10% heat-inactivated fetal calf serum (iFCS; Gibco, Thermo Fisher Scientific, Waltham, MA, USA), 100 U/mL penicillin/streptomycin (PAN-Biotech), 50 µM β-mercaptoethanol (β-Me; Gibco) and 2 mM L-Glutamine (L-Glut; PAN-Biotech). After 3 days, 10 mL R10 media supplied with 20 ng/mL mouse GM-CSF was added, and half of the media was replaced after 6 days. On day 8, non-adherent immature BMDCs (iBMDCs) were collected and matured for 24 h at a concentration of 0.7 x 10^6^ cells/mL in R10 media supplied with 20 ng/mL mouse GM-CSF and 100 ng/mL lipopolysaccharide (LPS) from E. coli O111:B4 (L4391; Sigma-Aldrich, St. Louis, MO, USA), revealing LPS-matured BMDCs (mBMDCs).

For phenotyping of immature and LPS-matured BMDCs, the following anti-mouse antibodies were used: PE-conjugate anti-CD11c (clone N418; BioLegend; #117308), BV421-conjugated anti-I-A/I-E (MHC-II; clone M5/114.15.2; BioLegend; #107632), APC-conjugated anti-CCR7 (clone 4B12; eBioscience; Thermo Fisher Scientific; #17-1971-82), APC-conjugated rat IgG2a kappa isotype control (clone eBR2a; eBioscience; Thermo Fisher Scientific; #17-4321-81), APC-conjugated anti-CD86 (clone GL-1; BioLegend; #105012), APC-conjugated anti-CD80 (clone 16-10A1; BioLegend; #104713) and APC-conjugated anti-CD40 (clone 3/23; BioLegend; #124612).

### 2.4 Generation of Flt3L-BMDCs for analysis of DC subsets

Bone marrow cells were isolated from femurs and tibias of mice, single cell suspensions were prepared and depleted of red blood cells using RBC lysing buffer. Subsequently, 15 x 10^6^ cells were seeded in 94 mm petri dishes in 10 mL R10 media supplemented with 100 ng/mL mouse Fms-related tyrosine kinase 3 ligand (Flt3L) from conditioned supernatant of a B16-Flt3L cell line (determined by ELISA). After 3 days, 10 mL R10 media supplied with 100 ng/mL Flt3L was added, and half of the media was replaced after 6 days. On day 8, non-adherent Flt3L-BMDCs were collected and DC subsets analyzed by flow cytometry. DC subsets were defined as follows: plasmacytoid DCs (pDCs): CD11c^+^CD11b^-^B220^+^; conventional DC1s (cDC1s): CD11c^+^B220^-^MHC-II^+^XCR1^+^ and conventional DC2s (cDC2s): CD11c^+^B220^-^MHC-II^+^CD172a^+^CD11b^+^. Cells were incubated with TruStain FcX (BioLegend) for 20 min at 4 °C to block Fc receptors, followed by surface staining for 20 min at 4 °C in Brilliant Stain Buffer (BD Biosciences) using the following antibodies: PE/Cy7-conjugated anti-mouse B220 (clone RA3-6B2; BioLegend; #103221), APC/Fire 750-conjugated anti-mouse I-A/I-E (MHC-II; clone M5/114.15.2; BioLegend; #107651), PE/Dazzle 594-conjugated anti-mouse CD11c (clone N418; BioLegend; #117347), PerCP-e710-conjugated anti-mouse CD172a (clone P84; eBioscience; # 46-1721-80), BV605-conjugated anti-mouse CD11b (clone M1/70; BioLegend; #101257) and BV786-conjugated anti-mouse XCR1 (clone ZET; BioLegend; #148225). Following surface staining, cells were incubated with Zombie Aqua dye (BioLegend) in PBS for 15 min at room temperature for live/dead discrimination.

### 2.5 Isolation of human primary cells and generation of human monocyte-derived (Mo)DCs

Blood donation for research purposes was approved by the local ethics committee and written consent was obtained from all donors. Human primary MoDCs were generated as described previously (37). Briefly, peripheral blood mononuclear cells were isolated by density gradient centrifugation using Ficoll-Paque Plus (Cytiva, Marlborough, MA, USA). Monocytes were then isolated by magnetic-activated cell sorting (MACS) using CD14 microbeads (Miltenyi Biotec, Bergisch Gladbach, Germany; #130-050-201) and differentiated for 6 days into immature MoDCs (iMoDCs) in serum-free AIM-V medium (Gibco) supplemented with human GM-CSF (50 ng/mL; PeproTech) and human IL-4 (50 ng/mL; PeproTech). On day 6, iMoDCs were collected and matured for 48h to generate mature MoDCs (mMoDCs) in GM-CSF/IL-4 containing media supplemented with a cytokine cocktail comprising human TNFα (20 mg/mL; PeproTech), human IL-6 (20 ng/mL; PeproTech) and human IL-1β (10 ng/mL; PeproTech).

### 2.6 Cell stimulation, preparation of lysates and Western blotting

To assess Spry2 protein expression, immature and LPS-matured BMDCs, as well as immature and mature MoDCs, were analyzed. Immature and LPS-matured BMDCs were enriched for CD11c^+^ cells by MACS using mouse CD11c microbeads (Miltenyi Biotec; #130-125-835). Cells were lysed for 30 min on ice in NP-40 buffer (20 mM Tris/HCl (pH 7.6), 50 mM NaCl, 10 mM MgCl_2_ and 1%Nonidet™ P 40 Substitute (Sigma-Aldrich)) containing cOmplete™ mini ethylenediaminetetraacetic acid (EDTA)-free protease inhibitor cocktail (Roche) and subsequently centrifuged at 20’000 x g for 30 min at 4 °C. Cleared lysates were mixed with 5 x sodium dodecyl sulfate (SDS) gel sample buffer (225 mM Tris–HCl (pH 6.8), 50% glycerol, 5% SDS, 4% β-Me, 0.03% bromophenol blue) and boiled at 95 °C for 5 min.

To elucidate phosphorylation of ERK1/2 and AKT upon CCL19 stimulation, LPS-matured BMDCs were stimulated with 50 nM human CCL19 (produced in house (55)) for indicated time periods. Stimulation was stopped by adding 5 x SDS gel sample buffer followed by vortexing and boiling at 95 °C for 5 min.

Proteins were separated on standard 10% Laemmli SDS gels and transferred to nitrocellulose membranes (Amersham™ Protran^®^, 0.45 µm; Cytiva, Marlborough, MA, USA) using semi-dry transfer units as described (56). Membranes were blocked in either 1 x ROTI-block (Carl ROTH, Karlsruhe, Germany) or 5% bovine serum albumin (BSA, Carl ROTH) in phosphate-buffered saline (PBS)-Tween (0.02%, PBST) at room temperature for 1 h followed by overnight incubation at 4 °C with the following primary antibodies (all from Cell Signaling Technology, Danvers, MA, USA): rabbit anti-Spry2 (#14954), rabbit anti-β-tubulin (#2146), rabbit anti-phospho-p44/42 MAPK (pERK1/2; Thr202/Tyr204; #4376), rabbit anti-p44/42 MAPK (tERK1/2; #9102), rabbit anti-phospho-AKT (pAKT; Ser473; #9271) and rabbit anti-AKT (tAKT; #9272). All antibodies were diluted in PBST containing 5% BSA and 0.02% NaN_3_. After washing with PBST, membranes were incubated with HRP-conjugated secondary antibodies (Jackson ImmunoResearch, West Grove, PA, USA) diluted 1:4000 in 5% dry milk and developed using Clarity™ Western ECL Substrate (Bio-Rad). Densiometric analysis was performed using the Image Lab software (Bio-Rad) by normalizing band volume intensities to β-tubulin, tERK1/2 or tAKT, respectively.

### 2.7 Flow cytometry

Staining of surface antigens was performed in staining buffer (PBS, 2% FCS, 2 mM EDTA) containing indicated antibodies for 20 min at 4 °C if not indicated otherwise. According to the manufacturer’s instructions, the Foxp3 transcription factor staining buffer set (Invitrogen, Thermo Fisher Scientific) was used for intracellular staining of transcription factors and the BD Cytofix/Cytoperm kit (BD Biosciences, San Jose, CA, USA) was used for intracellular cytokine staining. Samples were measured on a BD LSRII or LSRFortessa flow cytometer using the BD FACSDiva v6/9 software. Data was analyzed and illustrated using the FlowJo software (v10.9; BD Life Sciences, San Jose, CA, USA).

### 2.8 2D Transwell migration assay

LPS-matured BMDCs (1 x 10^5^ in 100 µL R10 medium) were seeded into the upper chamber on a 5 µm pore-sized polycarbonate membrane in a 24-well Transwell plate (Corning Costar; Corning, NY, USA). Cells were allowed to migrate towards the lower chamber wells containing 600 µL of R10 medium without chemokine (random migration) or containing graded concentrations of human CCL19 (0.02 nM - 2 µM). After a 3 h incubation at 37 °C, 5% CO_2_, migrated cells in the lower compartments were collected and cell numbers determined by flow cytometry. Sytox Blue Dead Cell Stain (Thermo Fisher) was used to exclude dead cells from the analysis. Specific migration was calculated as follows: % of specific migration = [(No. of migrated cells towards chemokine – No. of randomly migrated cells)/No. of cells in the input] *100.

### 2.9 3D collagen migration assay

Migration of LPS-matured BMDCs through a 3D collagen matrix was performed in ibiTreat µ-slide chemotaxis chambers (ibidi GmbH, Gräfelfing, Germany) as described previously (57, 58). Briefly, LPS-matured BMDCs were collected and resuspended at a concentration of 9 x 10^6^ cells/mL in R10 media. A mixture of 20 µL 10×DMEM, 10 µL 7.5% NaHCO_3_ and 150 µL PureCol collagen I (Advanced Biomatrix, Carlsbad, CA, USA) was prepared and carefully mixed with 90 µL cell suspension. 6.5 µL of the mixture was added to the observation area of the µ-slide chemotaxis chambers and allowed to polymerize for 45 min at 37 °C, 5% CO_2_. By adding media containing 100 nM human CCL19 to the right reservoir and media without chemokine to the left reservoir, a stable chemokine gradient was established (59). DC migration was monitored by time-lapse video microscopy using a 10x objective and a MRm camera on a Zeiss Axiovert 200M equipped with a Tokai Hit INU incubation system (Tokai Hit Co, Shizuoka, Japan) at 2 min intervals for 6 h at 37 °C. Single cells were tracked using the ‘Manual Tracking Plugin’ from Fiji/ImageJ and migration tracks were illustrated and quantified using the ‘Chemotaxis and Migration Tool’ software provided by ibidi.

### 2.10 Quantification of DC subsets in spleen and lymph nodes (LNs)

Analysis of DC subsets in spleen and LNs was performed using a protocol adapted from previously published protocols (60, 61). DC subsets in the spleen were defined as follows: pDCs: CD45^+^CD3ϵ^-^CD19^-^CD90^-^Ly6G^-^Ter119^-^NKp46^-^MerTK^-^B220^+^PDCA-1^+^Ly6C^+^; cDC1s: CD45^+^CD3ϵ^-^CD19^-^CD90^-^Ly6G^-^Ter119^-^NKp46^-^MerTK^-^B220^-^PDCA-1^-^Ly6C^-^CD11c^+^MHC-II^+^XCR1^+^CD172a^-^; cDC2s: CD45^+^CD3ϵ^-^CD19^-^CD90^-^Ly6G^-^Ter119^-^NKp46^-^MerTK^-^B220^-^PDCA-1^-^Ly6C^-^CD11c^+^MHC-II^+^XCR1^-^CD172a^+^; DC subsets in skin-draining LNs were defined as follows: pDCs: CD45^+^CD3ϵ^-^CD19^-^CD90^-^Ly6G^-^Ter119^-^NKp46^-^MerTK^-^B220^+^PDCA-1^+^Ly6C^+^; resident (res) cDC1s: CD45^+^CD3ϵ^-^CD19^-^CD90^-^Ly6G^-^Ter119^-^NKp46^-^MerTK^-^B220^-^PDCA-1^-^Ly6C^-^CD11c^high^MHC-II^int^XCR1^+^CD172a^-^; resident (res) cDC2s: CD45^+^CD3ϵ^-^CD19^-^CD90^-^Ly6G^-^Ter119^-^NKp46^-^MerTK^-^B220^-^PDCA-1^-^Ly6C^-^CD11c^high^MHC-II^int^XCR1^-^CD172a^+^; migratory (mig) cDC1s: CD45^+^CD3ϵ^-^CD19^-^CD90^-^Ly6G^-^Ter119^-^NKp46^-^MerTK^-^B220^-^PDCA-1^-^Ly6C^-^CD11c^int^MHC-II^high^XCR1^+^CD172a^-^; migratory (mig) cDC2s: CD45^+^CD3ϵ^-^CD19^-^CD90^-^Ly6G^-^Ter119^-^NKp46^-^MerTK^-^B220^-^PDCA-1^-^Ly6C^-^CD11c^int^MHC-II^high^XCR1^-^CD172a^+^EpCAM^-^. Markers for CD3ϵ, CD19, CD90, Ly6G, Ter119, NKp46 and MerTK were used as a lineage exclusion (lin) cocktail.

Spleens were harvested from mice, mechanically dissected into pieces and enzymatically digested in a solution containing 150 U/mL collagenase IV (Gibco), 40 µg/mL DNAse I (Roche) and 3 mM CaCl_2_ in R10 media for 30 min at 37 °C. Digested tissues were passed through a 70 µm cell strainer and the resulting single cell suspension was depleted of red blood cells using RBC lysing buffer.

Axial, brachial and inguinal LNs were harvested and digested as described above for the spleens and subsequently applied to a 70 µm cell strainer to obtain a single cell suspension. Cells were incubated with TruStain FcX (BioLegend) for 20 min at 4 °C to block Fc receptors. Surface staining was then performed for 30 min at 4 °C in Brilliant Stain Buffer (BD Biosciences) using the following antibodies: BV605-conjugated anti-mouse CD45 (clone 30-F11; BioLegend; #103139); APC-conjugated anti-mouse CD3ϵ (clone 145-2C11; BioLegend; #100311); APC-conjugated anti-mouse CD19 (clone 1D3; BioLegend; #152409); APC-conjugated anti-mouse CD90 (clone W20280E; BioLegend; #166405); APC-conjugated anti-mouse Ly6G (clone 1A8; BioLegend; #127613); APC-conjugated anti-mouse Ter119 (TER-119; BioLegend; #116211); APC-conjugated anti-mouse NKp46 (clone 29A1.4; BioLegend; #137607); APC-conjugated anti-mouse MerTK (clone 2B10C42; BioLegend; #151507); PE/Cy7-conjugated anti-mouse B220 (clone RA3-6B2; BioLegend; #103221); BV786-conjugated anti-mouse CD317 (PDCA-1; clone 927; BD Biosciences; #747603); Spark Plus UV395-conjugated anti-mouse Ly6C (clone HK1.4; BioLegend; #128073); APC/Fire 750-conjugated anti-mouse I-A/I-E (MHC-II; clone M5/114.15.2; BioLegend; #107651); PE/Dazzle 594-conjugated anti-mouse CD11c (clone N418; BioLegend; #117347); PerCP-e710-conjugated anti-mouse CD172a (clone P84; eBioscience; # 46-1721-80); and BV421-conjugated anti-mouse XCR1 (clone ZET; BioLegend; # 148216). For LN samples, PE-conjugated anti-mouse CD326 (EpCAM; clone G8.8; BD Biosciences; #563477) was additionally included. Following surface staining, cells were incubated with Zombie Aqua dye (BioLegend) in PBS for 15 min at room temperature for live/dead discrimination.

### 2.11 LN homing assay

LN homing assays were performed as described previously (54). LPS-matured Spry2^+/+^ and Spry2^-/-^ BMDCs were labeled with either CellTracker Deep Red (CTDR; 150 nM; Invitrogen, Thermo Fisher Scientific) or CellTracker Green (CTG; 4 µM; Invitrogen, Thermo Fisher Scientific) according to the manufacturer’s protocol. Dyes were swapped between the two groups within the same experiment to minimize potential dye-induced artifacts. Labeled Spry2^+/+^ and Spry2^-/-^ mBMDCs were mixed at a 1:1 ratio and a total of 2 x 10^6^ cells were injected into the footpad of the right lower extremity of recipient C57BL/6J mice anaesthetized with isoflurane. 24 h post-injection, draining and contralateral popliteal LNs (dPLNs and clPLNs) were harvested and digested as described above. Single cell suspensions were prepared using a 70 µm cell strainer and stained with PE-conjugated anti-mouse CD11c (clone N418; BioLegend; #117308), BV421-conjugated anti-mouse I-A/I-E (MHC-II; clone M5/114.15.2; BioLegend; #107632) and Fixable Viability Stain 575V (BD Bioscience) and analyzed by flow cytometry to determine the frequencies of mBMDCs that had migrated to dPLNs.

### 2.12 DC emigration from ear skin explants (ear crawl-out assay)

For the ear crawl-out assay, ears were harvested from *Spry2^+/+^CD11c-Cre^+^* (Spry2^+/+^) and *Spry2^fl/fl^CD11c-Cre^+^* (Spry2^-/-^) mice and subsequently split into ventral and dorsal halves. Explants were incubated for 72 h floating with the dermis side downwards on R10 media supplemented with 25 mM HEPES, 20 nM human CCL19 and 20 nM human CCL21 (produced in house) at 37 °C and 5% CO_2_. Emigrated cells were collected, stained with APC-conjugated anti-mouse CD11c (clone N418; BioLegend; #117310) and PE-conjugated anti-mouse I-A/I-E (MHC-II; clone M5/114.15.2; BioLegend; #107608) and TO-PRO-3 iodide to exclude dead cells and analyzed by flow cytometry.

To quantify the frequency of CD11c^+^MHC-II^+^ DCs in the skin, ears were harvested and split into ventral and dorsal halves. Skin explants were incubated in DMEM media containing 0.25 mg/mL liberase TL (Roche) and smashed through a 70 µm cell strainer. To quantify the percentage of DCs, prepared cell suspensions were stained with PE-conjugated anti-mouse CD11c (clone N418; BioLegend; #117308), BV421-conjugated anti-mouse I-A/I-E (MHC-II; clone M5/114.15.2; BioLegend; #107632) and TO-PRO-3 iodide (Invitrogen, Thermo Fisher Scientific) and subsequently analyzed by flow cytometry.

### 2.13 FITC skin painting assay

*Spry2^+/+^CD11c-Cre^+^* (Spry2^+/+^) and *Spry2^fl/fl^CD11c-Cre^+^* (Spry2^-/-^) mice were anaesthetized with isoflurane and 80 µL of a 1% FITC-solution (isomer I; Sigma-Aldrich; F7250) prepared in a 1:1 solution of acetone/dibutylphtalate (DBP; Sigma-Aldrich; #524980) was applied to the shaved abdomen of mice to induce skin irritation. After 24 h, draining axial and brachial LNs were harvested, digested and a single cell suspension was obtained as described above. Cells were stained with BV421-conjugated anti-mouse CD11c (clone N418; BioLegend; #117343) and AF647-conjugated anti-mouse I-A/I-E (MHC-II; clone M5/114.15.2; BioLegend; #107618) and evaluated by flow cytometry to determine the frequencies of immigrated CD11c^+^MHC-II^high^FITC^+^ DCs in the LNs.

### 2.14 FITC-dextran uptake assay

Immature BMDCs were collected on day 8 of the culture and 2 x 10^6^ cells per tube were stimulated with 100 ng/mL LPS for 30 min at 37 °C, 5% CO_2_. BMDCs were then incubated with 1 mg/mL FITC-dextran (FD40, Sigma-Aldrich) for indicated periods of time at 37 °C, 5% CO_2_. Uptake of FITC-dextran was stopped by adding ice-cold R10 media followed by two additional washing steps with ice-cold R10 media. Cells were resuspended in PBS, stained with TO-PRO-3 iodide and the uptake of FITC-dextran was quantified by flow cytometry.

### 2.15 T-cell proliferation assay

CD8^+^ T cells were purified from spleens of OT-I mice using the MojoSort™ Mouse CD8 T-Cell Isolation Kit (BioLegend), maintained overnight in R10 media supplemented with 40 U/mL recombinant murine IL-7 (PeproTech) and labeled with CellTrace Violet (CTV; 5 µM; Invitrogen; Thermo Fisher Scientific). LPS-matured BMDCs were pulsed with 0.1 or 1 µM SIINFEKL (Sigma-Aldrich; #S7951) for 2 h followed by a co-culture of 5 x 10^3^ DCs with 1 x 10^5^ CTV-labeled OT-I T cells for 72 h. Cells were stained using APC-conjugated anti-mouse CD8α (clone 53-6.7; BioLegend; #100712), and PE-conjugated anti-mouse Vα2 (clone B20.1; BioLegend; #127808), FITC-conjugated anti-mouse CD25 (clone 3C7; BioLegend; #101907) to assess T-cell activation. Based on CTV dilution profiles of CD8^+^Vα2^+^ T cells, division and proliferation indices were determined. The division index was calculated as ‘the total number of divisions’ divided by ‘the number of all cells of the original population’. The proliferation index was calculated as ‘the total number of divisions’ divided by ‘the number of cells that divided’. The parental peak represents CTV fluorescence intensity of undivided CD8^+^ T cells.

### 2.16 Infection of mice with LCMV-WE

LCMV-WE was originally obtained from F. Lehmann-Grube (Heinrich Pette Institute, University of Hamburg, Hamburg, Germany) and propagated on the fibroblast line L929. *Spry2^+/+^CD11c-Cre^+^* (Spry2^+/+^) and *Spry2^fl/fl^CD11c-Cre^+^* (Spry2^-/-^) mice were infected with 200 PFU LCMW-WE intravenously (*i.v.*) into the lateral tail vein. Mice were sacrificed 8 days after infection and spleen and LNs (axial, brachial, popliteal and inguinal) were harvested. Spleen and LNs were applied to a 70 µm cell strainer to obtain single cell suspensions. The splenocyte suspension was further depleted of red blood cells using RBC lysing buffer. Cells were stained for surface markers and transcription factors as described above using the following anti-mouse antibodies: BV421-conjugated anti CD8α (clone 53-6.7; BioLegend; #100753), FITC-conjugated anti CD3ϵ (clone 145-2C11; BioLegend; #100306), APC-conjugated anti CD44 (clone IM7; BioLegend; #103012), FITC-conjugated anti CD62L (MEL-14; BioLegend; #104406), APC-conjugated anti KLRG1 (clone 2F1/KLRG1; BioLegend; #138412), PE-conjugated anti CD127 (IL-7Rα; clone A7R34; BioLegend; #135010), APC-conjugated anti T-bet (clone 4B10; BioLegend; #644814) and PE-conjugated anti Eomes (clone Dan11mag; Invitrogen; Thermo Fisher Scientific; #12-4875-82).

For intracellular cytokine staining, cell suspensions prepared from spleen or LNs were plated in 96-well plates. Cells were incubated with or without 1 µM of the synthetic peptide GP_33–41_ (KAVYNFATC; obtained from P. Henklein, Charité, Berlin, Germany) for 4 h. 30 min after adding GP_33–41_, brefeldin A (Sigma-Aldrich; #B6542) was added to a final concentration of 10 µg/mL. Surface staining was performed using BV421-conjugated anti CD8α (clone 53-6.7; BioLegend; #100753) and FITC-conjugated anti CD3ϵ (clone 145-2C11; BioLegend; #100306), followed by intracellular staining with APC-conjugated anti-mouse IFNγ (clone XMG1.2; BD Biosciences; #562018), as described above.

### 2.17 Statistical analysis

Statistical analyses were performed using GraphPad Prism software v10.5.0 (GraphPad Software, San Diego, CA, USA). Unpaired or paired Student’s t-test were used to compare two datasets. Ordinary two-way analysis of variance (ANOVA) with Tukey’s or Šídák’s multiple comparisons test was used for multiple comparisons. Statistical significance was indicated as follows: ns = not significant (p > 0.05), *p ≤ 0.05, **p ≤ 0.01, ***p ≤ 0.001.

## 3 Results

### 3.1 Normal *in vitro* and *in vivo* differentiation of Spry2-deficient DCs

**Figure 1:**
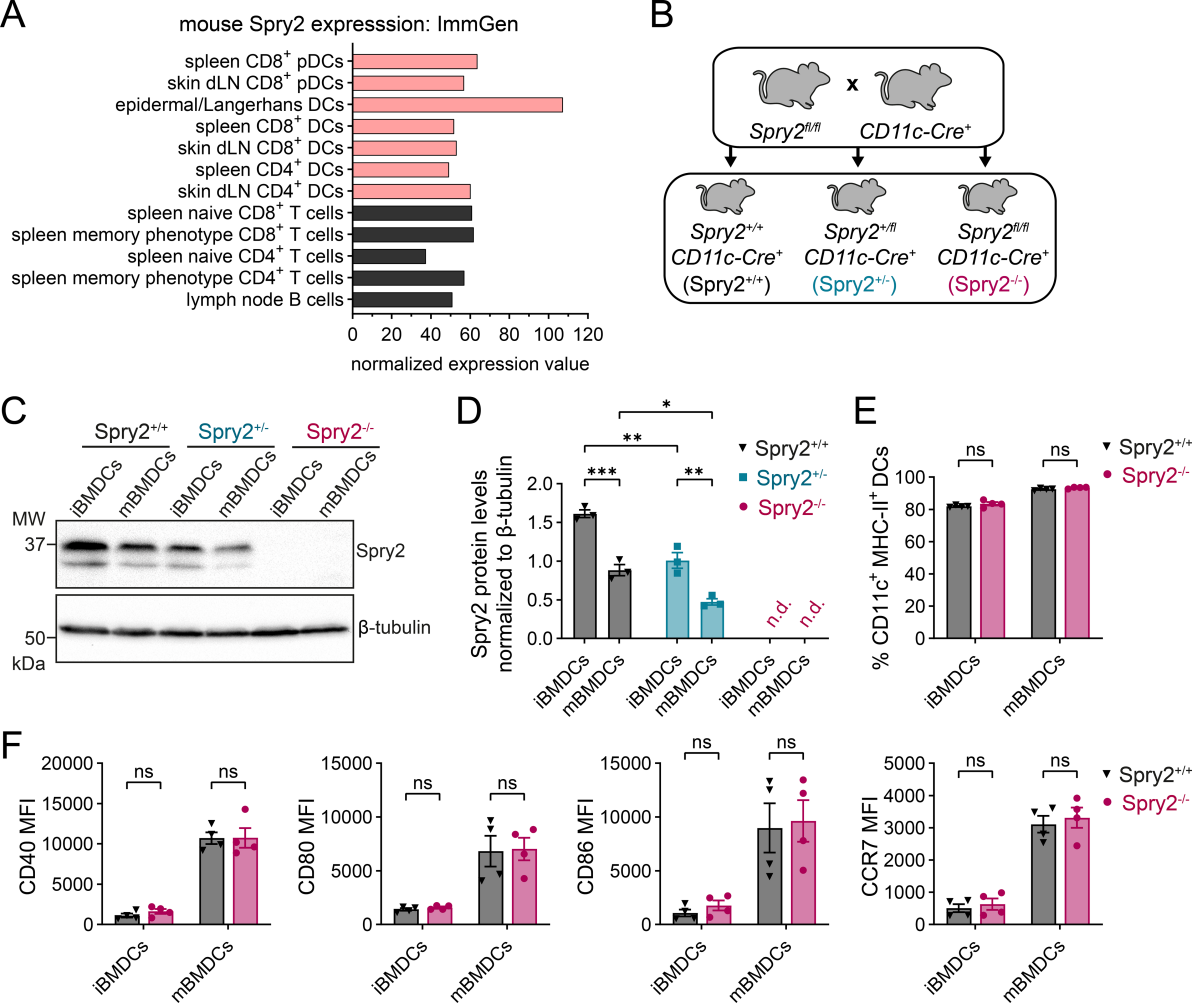
Normal differentiation and maturation of Spry2-deficient BMDCs. **(A)** Spry2 expression across mouse DC subsets and lymphocytes retrieved from the ImmGen database (ImmGen Microarray Gene Skyline; ProbeSet ID: 10422227; accessed January 16, 2026). **(B)** Generation of *Spry2^+/+^CD11c-Cre^+^* (Spry2^+/+^), *Spry2^+/fl^CD11c-Cre^+^* (Spry2^+/-^) and *Spry2^fl/fl^CD11c-Cre^+^* (Spry2^-/-^) mice by breeding *Spry2^fl/fl^* mice with hemizygous CD11c-Cre^+^ mice. For the Spry2 allele, ‘fl’ indicates a floxed allele and ‘+’ indicates a WT allele. For the CD11c-Cre transgene, ‘+’ indicates hemizygous expression. Abbreviations in brackets refer to the genotype of CD11c^+^ DCs and were used throughout the study. **(C, D)** Spry2 protein levels in immature (iBMDCs) and LPS-matured Spry2^+/+^, Spry2^+/-^ and Spry2^-/-^ BMDCs (mBMDCs) determined by Western blotting. **(C)** Representative Western blot showing Spry2 protein levels. **(D)** Quantification of Spry2 protein levels using densitometry. Bars show mean values ± SEM of BMDCs derived of 3 mice from 3 independent experiments; n.d., not detected. **(E, F)** BMDCs were generated by differentiating BM cells from Spry2^+/+^ and Spry2^-/-^ mice with GM-CSF for 8 days (iBMDCs), followed by LPS-induced maturation for 24 h (mBMDCs). Expression of characteristic surface markers was assessed by flow cytometry. **(E)** Frequencies of CD11c^+^MHC-II^int/high^ iBMDCS and CD11c^+^MHC-II^high^ mBMDCs (for gating strategy and representative plots see **Supplementary Figure S2A**). **(F)** Quantification of median fluorescence intensity (MFI) of CD40, CD80, CD86 and CCR7 surface expression on CD11c^+^MHC-II^+^ iBMDC and mBMDC populations (for representative plots see **Supplementary Figure S2B**). Bars show mean ± SEM of BMDCs derived from 4 mice in 4 independent experiments. Statistical differences were determined using ordinary two-way ANOVA with Tukey’s multiple comparisons test **(D)** or Šídák’s multiple comparisons test **(E, F)**; ns = not significant (p > 0.05), *p ≤ 0.05, **p ≤ 0.01, ***p ≤ 0.001.

The role of the ERK modulator Spry2 in DCs remains undefined so far. Analysis of mouse expression data retrieved from the ImmGen database revealed that Spry2 is expressed across multiple DC subsets at levels comparable to those in lymphocytes (**Figure 1A**). To investigate the relevance of Spry2 in DCs, we employed a genetic mouse model to delete Spry2 in DCs using the Cre-loxP system. Therefore, we crossed *Spry2^fl/fl^* mice (53) to *CD11c-Cre* mice (52), which hemizygously express the Cre recombinase transgene under the CD11c-Cre promoter, yielding *Spry2^fl/fl^CD11c-Cre^+^* mice with a CD11c-targeted knockout (KO) of Spry2 (**Figure 1B**; **Supplementary Figure S1A**). To generate BMDCs from *Spry2^+/+^CD11c-Cre^+^* (Spry2^+/+^), *Spry2^+/fl^CD11c-Cre^+^* (Spry2^+/-^) and *Spry2^fl/fl^CD11c-Cre^+^* (Spry2^-/-^) mice, BM cells were cultured in the presence of GM-CSF for eight days followed by lipopolysaccharide (LPS)-induced maturation. Western blot analysis of CD11c^+^-sorted immature (iBMDCs) and LPS-matured BMDCs (mBMDCs) confirmed efficient Spry2 deletion in Spry2^-/-^ BMDCs and revealed reduced Spry2 protein levels in Spry2^+/-^ compared to Spry2^+/+^ BMDCs (**Figures 1C, D**). Hence, Spry2^+/+^ mice were used as controls for subsequent experiments. Notably, LPS-induced maturation led to a downregulation of Spry2 in both Spry2^+/-^ and Spry2^+/+^ mBMDCs (**Figures 1C, D**). Consistently, examining data from the Human Protein Atlas (Monaco dataset (62)) revealed Spry2 expression in human myeloid and plasmacytoid DCs (**Supplementary Figure S1B**). Furthermore, Spry2 expression was detected in human monocyte-derived (Mo)DCs and was downregulated upon maturation (**Supplementary Figures S1C, D**), similar to our observations in mouse BMDCs. To determine whether Spry2 deficiency affects BMDC differentiation and maturation, we analyzed the expression of characteristic DC surface markers on BMDCs generated from Spry2^+/+^ and Spry2^-/-^ mice. BM progenitors from both genotypes generated comparable frequencies of CD11c^+^MHC-II^int/high^ iBMDCs and CD11c^+^MHC-II^high^ mBMDCs (**Figure 1E**; **Supplementary Figure S2A**). Additionally, expression levels of the co-stimulatory molecules CD40, CD80 and CD86 as well as the chemokine receptor CCR7 were low on iBMDCs and strongly upregulated upon LPS-maturation, with no discernible differences between Spry2^+/+^ and Spry2^-/-^ BMDCs (**Figure 1F**; **Supplementary Figure S2B**). Thus, GM-CSF-driven *in vitro* BMDC differentiation and LPS-induced maturation remain intact in the absence of Spry2.

**Figure 2:**
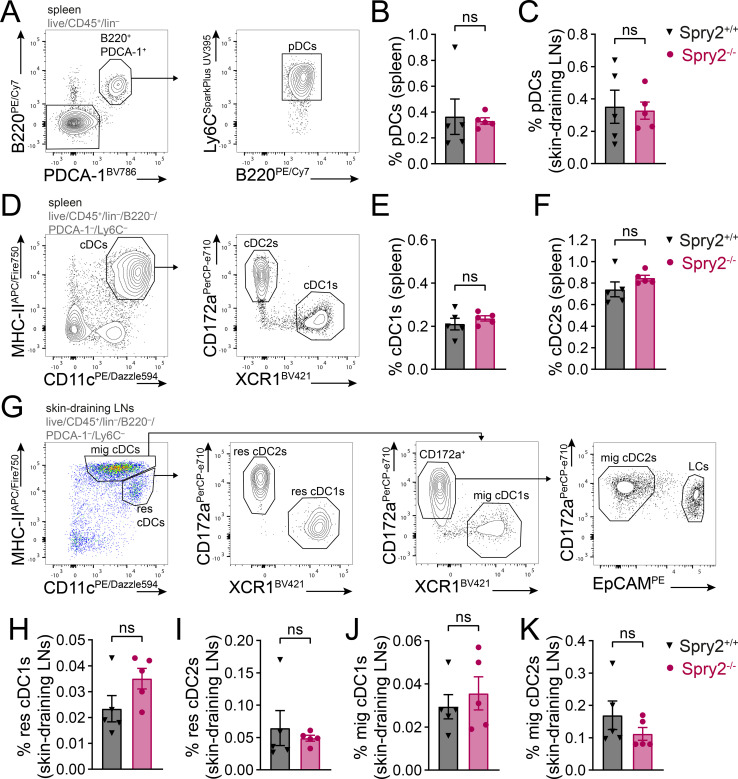
*In vivo* differentiation of DC subsets in spleen and skin-draining lymph nodes is unaffected by Spry2 deficiency in DCs under steady-state conditions. **(A-K)** Spleen and skin-draining LNs (axial, brachial and inguinal) from Spry2^+/+^ and Spry2^-/-^ mice were enzymatically digested using collagenase IV and DNase I and DC subsets were analyzed by flow cytometry. The lineage (lin) exclusion cocktail includes antibodies against CD3ϵ, CD19, CD90, Ly6G, Ter119, NKp46 and MerTK. **(A)** Gating strategy to identify plasmacytoid DCs (pDCs; CD45^+^lin^-^B220^+^PDCA-1^+^Ly6C^+^) in spleen and skin-draining LNs. Representative gating is shown for the spleen. Frequencies of pDCs within live CD45^+^ cells **(B)** in the spleen and **(C)** in skin-draining LNs. **(D)** Gating strategy to identify conventional DC1s (cDC1s; CD45^+^lin^-^B220^-^PDCA-1^-^Ly6C^-^CD11c^+^MHC-II^+^XCR1^+^CD172a^-^) and cDC2s (CD45^+^lin^-^B220^-^PDCA-1^-^Ly6C^-^CD11c^+^MHC-II^+^XCR1^-^CD172a^+^) in the spleen. Frequencies of **(E)** cDC1s and **(F)** cDC2s within live CD45^+^ cells in the spleen. **(G)** Gating strategy to identify resident (res) cDC1s (CD45^+^lin^-^B220^-^PDCA-1^-^Ly6C^-^CD11c^high^MHC-II^int^XCR1^+^CD172a^-^), res cDC2s (CD45^+^lin^-^B220^-^PDCA-1^-^Ly6C^-^CD11c^high^MHC-II^int^XCR1^-^CD172a^+^), migratory (mig) cDC1s (CD45^+^lin^-^B220^-^PDCA-1^-^Ly6C^-^CD11c^int^MHC-II^high^XCR1^+^CD172a^-^) and mig cDC2s (CD45^+^lin^-^B220^-^PDCA-1^-^Ly6C^-^CD11c^int^MHC-II^high^XCR1^-^CD172a^+^EpCAM^-^) in skin-draining LNs; LCs, Langerhans cells. Frequencies of **(H)** res cDC1s, **(I)** res cDC2s, **(J)** mig cDC1s and **(K)** mig cDC2s within live CD45^+^ cells in skin-draining LNs. Bars show mean ± SEM of 5 individual mice from 2 independent experiments. Statistical differences were determined using Student’s unpaired t-test **(B, C, E, F, H-K)**; ns = not significant (p > 0.05).

To determine whether Spry2 deficiency affects *in vivo* differentiation of distinct DC subsets, we analyzed DC populations in the spleen and skin-draining LNs. We found similar frequencies of pDCs in the spleen (**Figures 2A, B**) and skin-draining LNs (**Figure 2C**) of Spry2^+/+^ and Spry2^-/-^ mice. Likewise, the frequencies of conventional DC1s (cDC1s) and cDC2s in the spleen were not altered by Spry2 deficiency (**Figures 2D–F**). In skin-draining LNs, resident (res) cDC1 and cDC2 populations were present at comparable frequencies in Spry2^+/+^ and Spry2^-/-^ mice (**Figures 2G–I**). Moreover, the frequencies of migratory (mig) cDC1s and cDC2s were also similar between the two genotypes (**Figures 2G, J, K**). In line with this, Flt3L-driven differentiation of BM from Spry2^+/+^ and Spry2^-/-^ mice resulted in comparable frequencies of pDCs, cDC1s or cDC2s in Flt3L-BMDC cultures (**Supplementary Figures S2C–F**). Together, these data indicate that DC differentiation into distinct DC subsets is unaffected by Spry2 deficiency both *in vitro* and *in vivo.*

### 3.2 Efficient CCR7-driven migration and LN homing of Spry2-deficient mBMDCs

**Figure 3:**
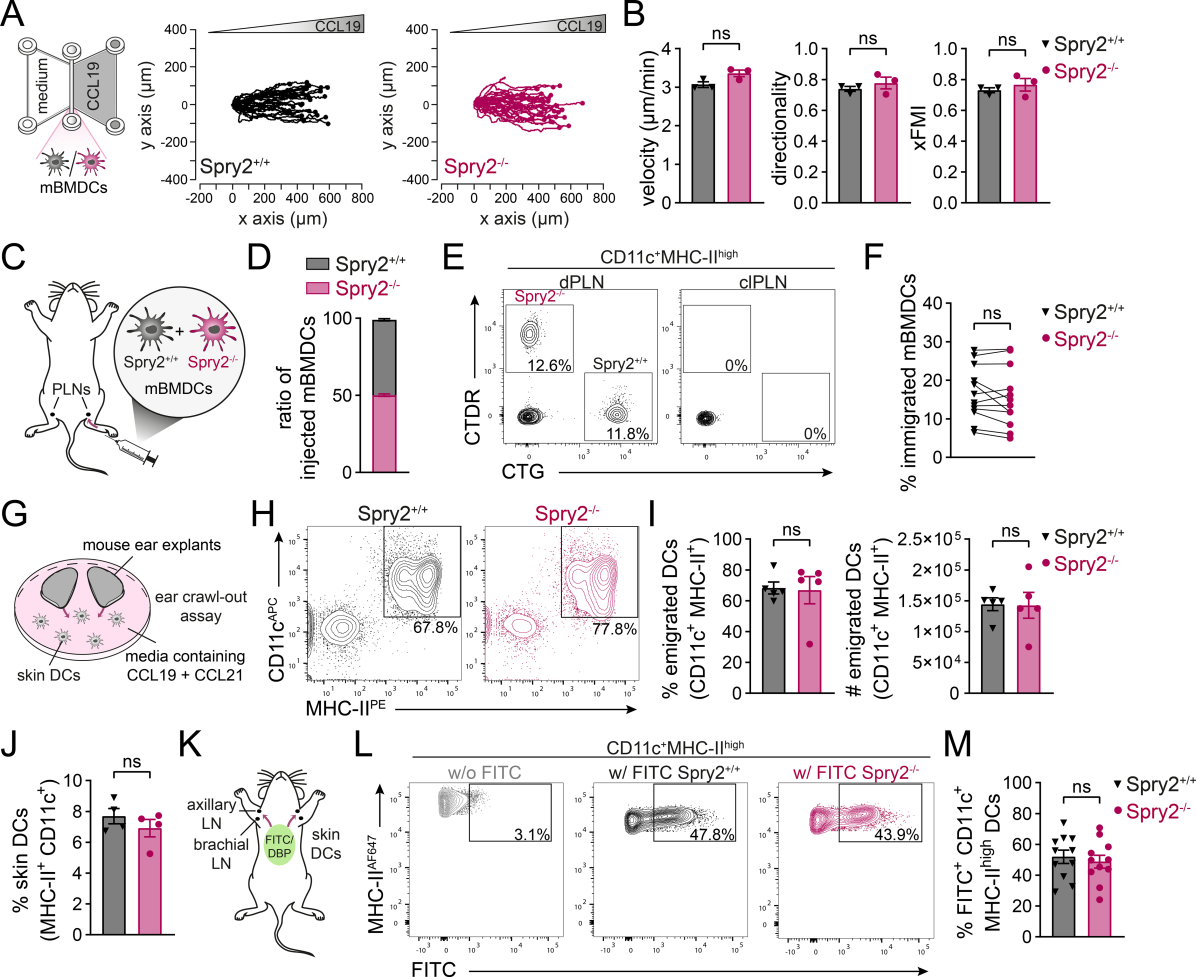
Spry2-deficient DCs show efficient CCR7-driven migration and *in vivo* LN homing. **(A, B)** Spry2^+/+^ and Spry2^-/-^ mBMDCs were embedded into a 3D collagen matrix using µ-slide chemotaxis chambers and allowed to migrate along CCL19 gradients. Cell migration was recorded by time-lapse video microscopy and cells were manually tracked. **(A)** Experimental setup using the µ-slide chemotaxis chamber (left panel) and migration tracks of 30 mBMDCs from one representative experiment centered on the same starting point (right panels). **(B)** Quantification of velocity, directionality and forward migration index (xFMI). Individual data points represent means of independent experiments, bars show mean ± SEM of 79 cells pooled from 3 independent experiments. **(C-F)** Spry2^+/+^ and Spry2^-/-^ mBMDCs were labeled with CellTracker Green (CTG) or CellTracker Deep Red (CTDR) and a 1:1 mixture was injected into the footpad of C57BL/6J recipient mice. Immigration of mBMDCs in draining and contralateral popliteal LNs (dPLNs and clPLNs) was determined after 24 h by flow cytometry. **(C)** Scheme of the LN homing assay. **(D)** Ratio of injected CTG or CTDR labeled mBMDCs. **(E)** Representative contour plots showing immigrated CTG- and CTDR-labeled mBMDC populations gated on live/CD11c^+^MHC-II^high^ migratory DCs in PLNs. **(F)** Frequencies of Spry2^+/+^ and Spry2^-/-^ mBMDCs in dPLNs, pre-gated on live/CD11c^+^MHC-II^high^ cells in dPLNs. Data points of 12 mice pooled from 3 independent experiments. **(G-I)** Emigration of skin DCs from Spry2^+/+^ and Spry2^-/-^ split ear explants floating on media containing CCL19 and CCL21 (20 nM each) for 72 h was quantified by flow cytometry. **(G)** Scheme of the ear crawl-out assay. **(H)** Representative contour plots depicting frequencies of CD11c^+^MHC-II^+^ DCs emigrated from skin explants. **(I)** Frequencies (% of live cells; left panel) and absolute numbers (right panel) of emigrated CD11c^+^MHC-II^+^ DCs. Bars show mean ± SEM from explants of 5 mice from at least 4 independent experiments. **(J)** Ear skin of Spry2^+/+^ and Spry2^-/-^ mice was digested using liberase TL and DC populations were analyzed by flow cytometry. Frequencies of CD11c^+^MHC-II^+^ DCs in the skin, pre-gated on live cells in the skin. Bars show mean ± SEM of 4 individual mice from 3–4 independent experiments. **(K-M)** Abdomen of Spry2^+/+^ and Spry2^-/-^ mice were shaved and a dibutylphtalate (DBP) containing FITC solution was applied. Frequencies of FITC^+^ skin DCs in dLNs (axial and brachial) were determined after 24 h by flow cytometry. **(K)** Scheme of the FITC skin painting assay. **(L)** Representative contour plots depicting FITC^+^ populations gated on CD11c^+^MHC-II^high^ migratory DCs in dLNs of unpainted control (w/o FITC) and FITC-painted (w/FITC) Spry2^+/+^ and Spry2^-/-^ mice. **(M)** Frequencies of FITC^+^CD11c^+^MHC-II^high^ DCs that migrated to dLNs (% within CD11c^+^MHC-II^high^ cells in the LN). Bars show mean ± SEM of 11 mice from 4 independent experiments. Statistical differences were determined using Student’s unpaired **(B, I, J, M)** and paired **(F)** t-test; ns = not significant (p > 0.05).

To elucidate the role of Spry2 in DC migration, Spry2^+/+^ and Spry2^-/-^ mBMDCs were first analyzed in a 2D Transwell migration assay, revealing similar migratory responses to graded concentrations of CCL19 (**Supplementary Figures S3A, B**). To monitor DC migration under more physiological conditions in a 3D environment, we used µ-slide chemotaxis chambers in which a stable chemokine gradient can be established (59). mBMDCs were embedded in a collagen matrix, exposed to a CCL19 gradient and migration was monitored by time-lapse microscopy. Consistent with our 2D migration results, Spry2^+/+^ and Spry2^-/-^ DCs efficiently migrated towards the chemokine source with similar speed, directionality and forward migration index (FMI) (**Figures 3A, B**). Concluding, these findings demonstrate that Spry2 deficiency does not alter the migratory capacity of DCs towards CCL19 under simplified 2D and more physiologically relevant 3D conditions.

The use of 3D collagen migration models already provides a more physiologically relevant approach to mimic the environment migrating cells encounter *in vivo*. However, *in vitro* reconstituted collagen networks are rather homogenous with uniform pore sizes and therefore incompletely represent the structural complexity of interstitial tissues and organs (63). Consequently, we aimed to investigate whether the loss of Spry2 affects DC migration *in vivo*. Therefore, we first assessed the *in vivo* LN homing capacity of Spry2^-/-^ mBMDCs. Spry2^+/+^ and Spry2^-/-^ mBMDCs were separately labeled with distinct fluorescent CellTracker dyes and co-injected as a 1:1 mixture into the footpads of C57BL/6J recipient mice (**Figures 3C, D**). After 24 h, we quantified the arrival of mBMDCs within the CD11c^+^MHC-II^high^ DC population in draining popliteal LNs (dPLNs), and as a negative control in contralateral PLNs (clPLNs; **Figure 3E**). Spry2^+/+^ and Spry2^-/-^ mBMDCs migrated to dPLNs with similar efficiencies and as expected, mBMDCs did not home to clPLNs (**Figures 3E, F**). Thus, we detect no discernible alterations in the *in vivo* LN homing potential of mBMDCs caused by Spry2 deficiency.

### 3.3 Effective migration of Spry2-deficient skin-resident DCs

Next, we extended our explorations to skin-resident DCs, including epidermal Langerhans cells and dermal DCs, using two different approaches. First, we performed an *ex vivo* crawl-out assay to investigate the emigration of DCs from ear skin explants. Therefore, ears were carefully split into ventral and dorsal halves and cutaneous DCs were allowed to crawl-out into media containing the chemokines CCL19 and CCL21 (**Figure 3G**). We then quantified the number of CD11c^+^MHC-II^+^ DCs that emigrated into the media and observed that both, Spry2^+/+^ and Spry2^-/-^ DCs, untaintedly migrated towards the chemokine source with similar efficacy (**Figures 3H, I**). As we found comparable frequencies of CD11c^+^MHC-II^+^ DC populations in the skin of Spry2^+/+^ and Spry2^-/-^ mice (**Figure 3J**), we conclude that Spry2 deficiency does not detectably alter the *ex vivo* migration of skin-resident DCs. Finally, we aimed to monitor CCR7-driven trafficking of DCs to skin-dLNs under inflammatory conditions in a FITC skin painting assay. To this end, the abdomen of Spry2^+/+^ and Spry2^-/-^ mice were shaved and epicutaneously treated with a FITC solution prepared in the skin irritant dibutylphthalate (DBP) to induce DC homing to dLNs (38) (**Figure 3K**). After 24 h, the percentage of CD11c^+^MHC-II^high^FITC^+^ migratory DCs in the axillary and brachial dLNs was evaluated by flow cytometry. Spry2^+/+^ and Spry2^-/-^ skin-resident DCs readily homed to the dLNs as demonstrated by a clearly identifiable population of migratory FITC^+^ DCs within dLNs (**Figure 3L**). Notably, the percentage of immigrated FITC^+^ DCs detected in dLNs was similar for Spry2^+/+^ and Spry2^-/-^ mice (**Figure 3M**), consistently demonstrating that the absence of Spry2 does not affect CCR7-dependent LN homing of cutaneous DCs *in vivo*.

### 3.4 Spry2-deficient DCs show efficient CCR7-mediated ERK activation, antigen uptake and CD8^+^ T-cell priming *in vitro*

**Figure 4:**
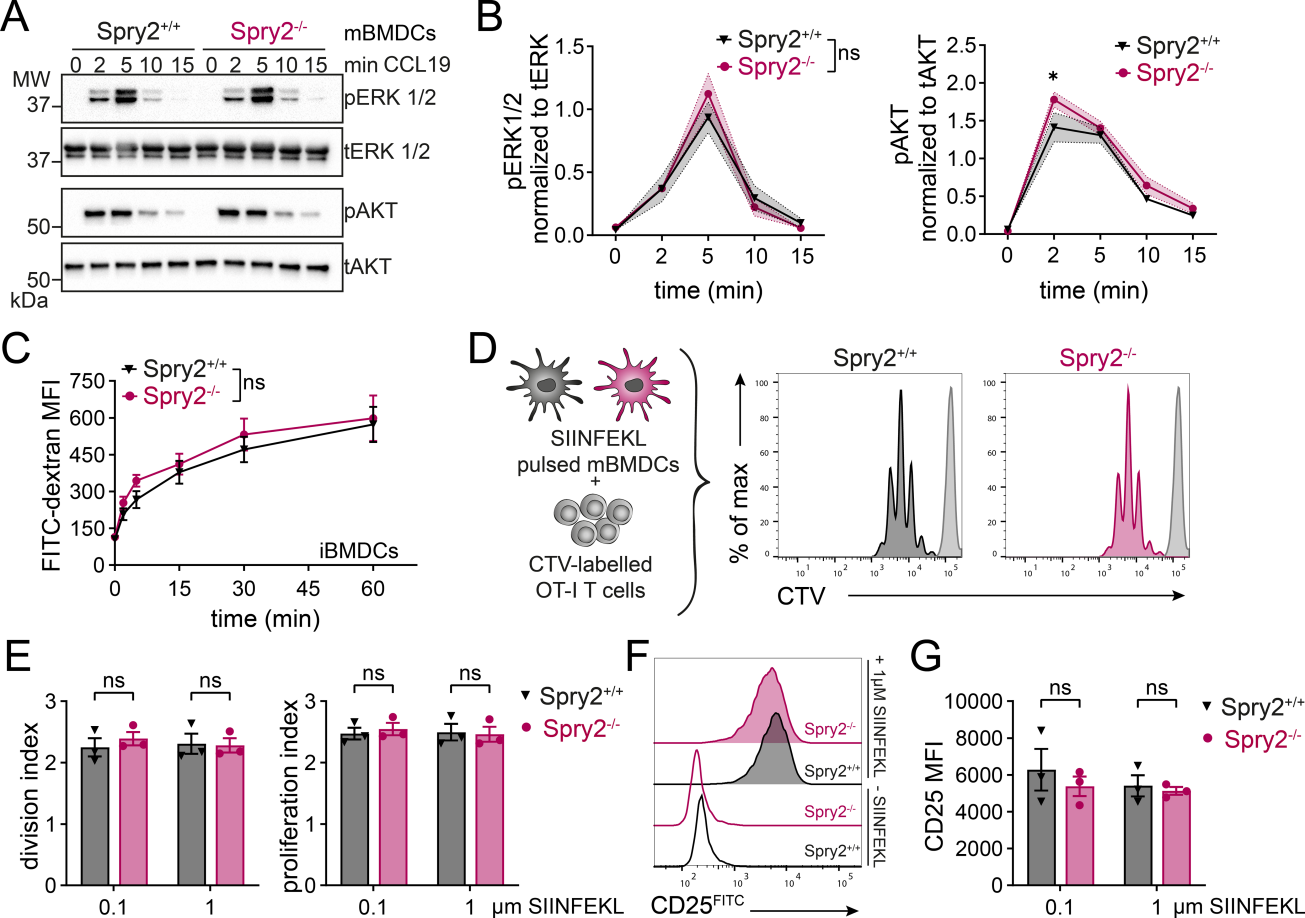
Spry2-deficient DCs show efficient CCR7-mediated ERK activation, antigen uptake and CD8^+^ T-cell priming *in vitro*. **(A, B)** Spry2^+/+^ and Spry2^-/-^ mBMDCs were stimulated with 50 nM CCL19 for indicated time periods. Total (t) and phosphorylated (p) protein levels of ERK1/2 and AKT were analyzed by Western blotting. **(A)** Representative Western blot showing pERK1/2, tERK1/2, pAKT and tAKT protein levels. **(B)** Quantification of pERK1/2 (left panel) and pAKT (right panel) protein levels normalized to total ERK1/2 and AKT, respectively. Mean values ± SEM of 4 independent experiments. **(C)** Spry2^+/+^ and Spry2^-/-^ iBMDCs were pre-stimulated with LPS for 30 min and subsequently incubated with a FITC-dextran solution (1 mg/mL) for indicated times. Median fluorescence intensity (MFI) determined by flow cytometry was used to quantify FITC-dextran uptake. Mean values ± SEM of 3 independent experiments. **(D-G)** Spry2^+/+^ and Spry2^-/-^ mBMDCs were pulsed with 0.1 or 1 µM SIINFEKL peptide and co-cultured with CellTrace Violet (CTV)-labeled OT-I T cells for 72 h. T-cell proliferation and activation was analyzed by flow cytometry. **(D)** Scheme of the *in vitro* T-cell proliferation assay (left panel) and representative proliferation profiles (right panels) with parental (undivided) peak shown in light gray. **(E)** Quantification of OT-I T-cell division (left panel) and proliferation (right panel) indices. **(F)** Representative histograms of CD25 expression on OT-I T cells co-cultured with either 1 µM SIINFEKL-pulsed or unpulsed mBMDCs. **(F)** Quantification of CD25 expression on OT-I T cells (mean fluorescence intensity, MFI). Bars show mean ± SEM of 3 independent experiments. Statistical differences were determined using ordinary two-way ANOVA with Šídák’s multiple comparisons test; ns = not significant (p > 0.05), *p ≤ 0.05.

Spry2 is a well-known modulator of ERK1/2 signaling, reported to exert both activating and inhibitory effects (5, 7, 8). CCR7 activation by cognate ligands triggers a cascade of downstream signaling events, including the phosphorylation of the key kinases ERK1/2 and AKT involved in DC migration and survival (47, 64–66). Stimulation of mBMDCs with CCL19 induced a transient phosphorylation pattern of both effector kinases with similar kinetics for Spry2^+/+^ and Spry2^-/-^ mBMDCs (**Figure 4A**). ERK1/2 phosphorylation peaked at 5 min post-stimulation with similar intensities, whereas phosphorylation of AKT peaked at 2 min and appeared slightly enhanced in Spry2^-/-^ mBMDCs (**Figure 4B**). Concluding, CCL19-stimulation induces efficient phosphorylation of ERK1/2 and AKT in Spry2^-/-^ mBMDCs.

DCs are not only specialized in migration but are also highly proficient in sampling, processing and presenting antigens to naive T cells to initiate an adaptive immune response. Since migratory abilities appeared normal, we further queried whether Spry2 deficiency affects antigen uptake. To this end, iBMDCs were pre-stimulated with LPS for 30 min and uptake of FITC-dextran was monitored over time. Spry2^+/+^ and Spry2^-/-^ iBMDCs exhibited similar uptake of FITC-dextran, which steadily increased over time (**Figure 4C**).

Subsequently, we compared the ability of Spry2^+/+^ and Spry2^-/-^ mBMDCs to prime cognate T cells by *in vitro* co-culturing SIINFEKL-pulsed mBMDCs with CellTrace Violet (CTV)-labeled OT-I CD8^+^ T cells for 72 h (**Figure 4D**). Both cell types were equally effective in CD8^+^ T-cell priming as indicated by similar proliferation profiles, T-cell division and proliferation indices (**Figures 4D, E**), as well as CD25 surface expression (**Figures 4F, G**), regardless of the SIINFEKL concentration. Hence, Spry2^-/-^ mBMDCs demonstrate an unrestricted capacity to prime CD8^+^ T cells *in vitro*.

### 3.5 Spry2-deficient DCs induce normal CD8^+^ T-cell effector differentiation during acute viral infection

**Figure 5:**
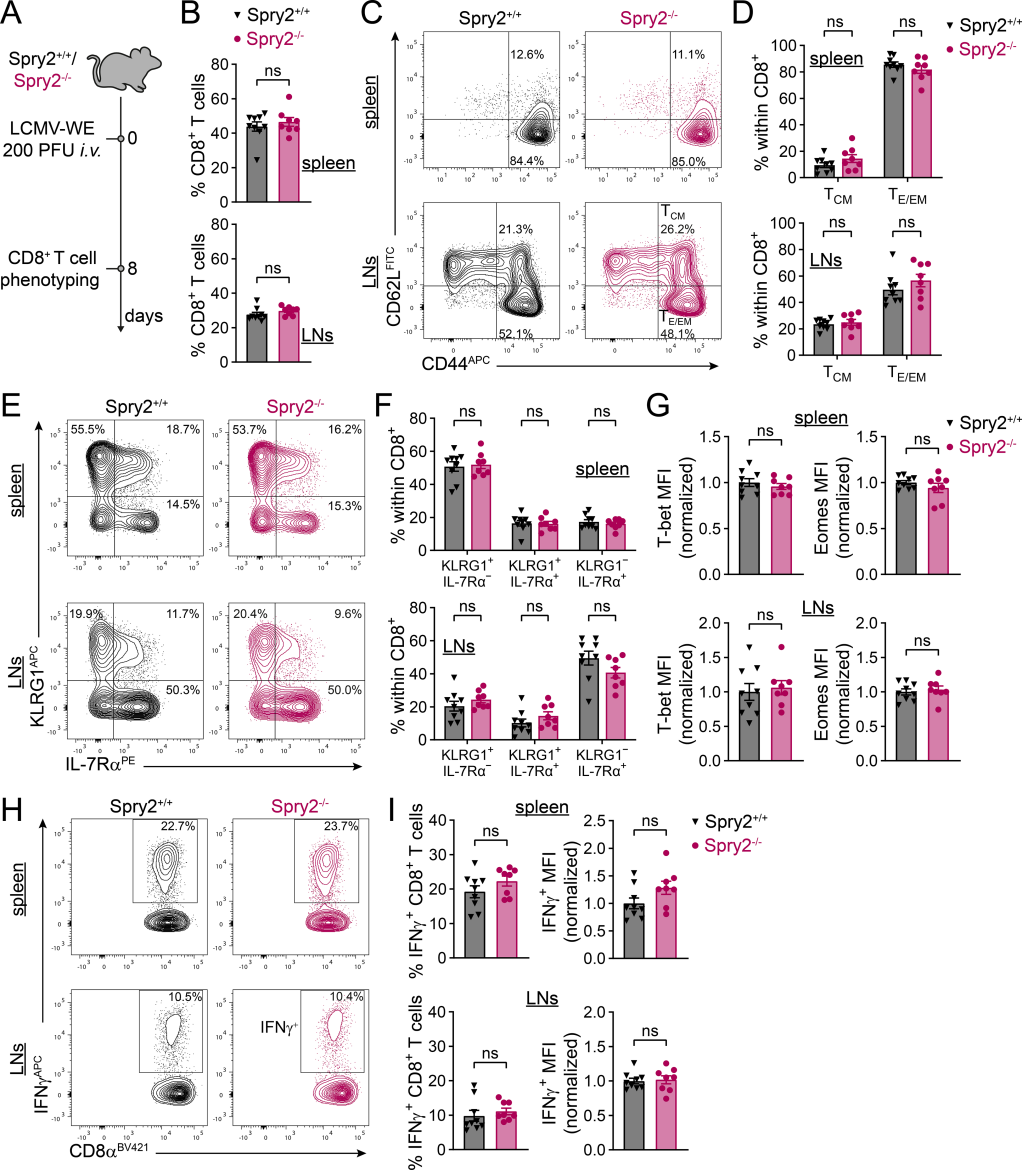
Spry2-deficient DCs induce normal CD8^+^ T-cell effector differentiation during acute viral infection. **(A-I)** Spry2^+/+^ and Spry2^-/-^ mice were infected with 200 PFU LCMV-WE (*i.v.*). CD8^+^ T cells in spleen (upper panels) and in axial, brachial, popliteal and inguinal LNs (lower panels) were analyzed 8 days post infection by flow cytometry. **(A)** Schematic overview of the LCMV infection experiment. **(B)** Frequencies of CD8^+^ T cells. **(C)** Representative contour plots of CD62L and CD44 expression on CD8^+^ cells to determine central memory (T_CM_) and effector/effector memory (T_E/EM_) T cells. **(D)** Frequencies of T_CM_ (CD62L^+^CD44^+^) and T_E/EM_ (CD62L^-^CD44^+^) subsets. **(E)** Representative contour plots depicting KLRG1 and IL-7Rα expression on CD8^+^ cells. **(F)** Frequencies of KLRG1^+^IL-7Rα^-^, KLRG1^+^IL-7Rα^+^ and KLRG1^-^IL-7Rα^+^ CD8^+^ T-cell populations. **(G)** Quantification of T-bet (left panel) and Eomes (right panel) expression of CD8^+^ T cells (normalized median fluorescence intensity, MFI). **(H)** Representative contour plots of IFNγ expression of CD8^+^ T cells after *ex vivo* restimulation with 1 µM LCMV-GP_31–44_ peptide for 4 h. **(I)** Frequencies (left panel) and normalized median fluorescence intensity (MFI; right panel) of IFNγ^+^CD8^+^ T cells after *ex vivo* restimulation. Bars show mean ± SEM of 8–9 mice from 2 independent experiments. Statistical differences were determined using Student’s unpaired t-test **(B, G, I)** and ordinary two-way ANOVA with Šídák’s multiple comparisons test **(D, F)**; ns = not significant (p > 0.05).

Effective immune protection against viruses requires the generation of phenotypically and functionally distinct effector and memory T-cell subsets. During infection, naive CD8^+^ T cells are primed by antigen-presenting DCs, rapidly expand and differentiate into diverse effector subsets, regulated by the expression of distinct transcription factors (TFs) and epigenetic changes (67, 68). A large proportion of cells become terminal effector (TE) cells, characterized by high killer cell lectin-like receptor G1 (KLRG1) and low IL-7 receptor subunit α (IL-7Rα) expression. These cells are highly cytotoxic and efficient in fighting the infection but mostly die during the contraction phase (67, 69, 70). In contrast, memory precursor (MP) cells, expressing low levels of KLRG1 and high levels of IL-7Rα, show enhanced survival and multipotency as they give rise to multiple types of memory cells important for long-term protective immunity (67, 71, 72). In addition, KLRG1 and IL-7Rα double positive (DP) cells become ex-KLRG1 cells during contraction and further differentiate into memory T cells that retain high cytotoxic capacity (73, 74). Factors that influence this cell fate decision include TCR signal strength and quality, co-stimulatory and co-inhibitory signals as well as cytokine stimulation during the process of T-cell priming (68). Therefore, we examined the effect of Spry2 deficiency in DCs on the CD8^+^ T-cell effector differentiation upon acute viral infection. To this end, we intravenously infected Spry2^+/+^ and Spry2^-/-^ mice with the lymphocytic choriomeningitis virus (LCMV)-WE and assessed the activation and differentiation status of the endogenous CD8^+^ T-cell populations in spleen and LNs at the peak of the cytotoxic T-cell response (**Figure 5A**). Eight days post infection, the frequencies of CD8^+^ T cells present in spleen and LNs of Spry2^+/+^ and Spry2^-/-^ mice were similar (**Figure 5B**). In addition, we found similar expression patterns of CD44 and CD62L, with the predominant population being CD8^+^CD44^+^CD62L^–^ effector/effector memory T cells (T_E/EM_) in spleen and LNs of both groups (**Figures 5C, D**). Furthermore, the frequencies of KLRG1^+^IL-7Rα^–^, KLRG1^+^IL-7Rα^+^ and KLRG1^–^ IL-7Rα^+^ CD8^+^ T-cell populations were similar in the spleen and LNs of Spry2^+/+^ and Spry2^-/-^ mice (**Figures 5E, F**). Two key TFs that shape T-cell fate toward TE or MP differentiation are T-bet and Eomes (69, 75, 76). Consistent with our previous findings, expression levels of T-bet and Eomes in CD8^+^ T cells did not differ between Spry2^+/+^ and Spry2^-/-^ mice (**Figure 5G**). Finally, to assess the functional capacity of CD8^+^ T cells, we quantified interferon (IFN)γ production upon *ex vivo* restimulation with the LCMV-specific peptide GP_33-41_. Both, the frequency of IFNγ-producing CD8^+^ T cells and the amount of IFNγ produced were similar between Spry2^+/+^ and Spry2^-/-^ mice (**Figures 5H, I**). In conclusion, lack of Spry2 seems to have no impact on the DCs capacity to induce effector differentiation of CD8^+^ T cells during an antiviral immune response.

## 4 Discussion

Controlled homing of mature DCs to dLNs and subsequent priming of cognate T cells is essential for initiating and shaping an effective adaptive immune response. Aberrant LN trafficking of DCs can lead either to impaired immune responses against pathogens or to unwanted immune activation resulting in autoimmunity. Consequently, targeting DC migration in therapeutic strategies holds significant potential, both for suppressing autoimmune reactions and for enhancing DC-based immunotherapies. Therefore, studying DC migration and identifying positive or negative regulators of DC function is essential for unraveling the complexity of their immune functions. Equally important is ruling out potential candidates that do not influence DC responses. Although this may be less relevant for therapeutic applications, it is important for advancing our understanding of the mechanisms that govern DC activity. Here, we investigated the role of Spry2 in DCs for the first time by generating a CD11c-targeted Spry2 KO mouse model. Spry2 is one of four members of the Spry protein family, which are best known for positively or negatively regulating the ERK signaling pathway in a highly context- and cell type-specific manner (5, 8). Its function in the immune system has only recently begun to emerge as Spry2 was shown to affect *in vitro* macrophage polarization (77) and to modulate B- and T-cell responses (27–31). In addition, human and mouse Spry2 have been reported to enhance (18, 23) or inhibit (12, 19, 20, 22, 24, 78) cell migration of non-immune cells through both ERK-dependent and ERK-independent mechanisms. In line with its multifaceted nature, Spry2 seems to regulate migration through various mechanisms that require further investigation. For example, early findings suggest that the anti-migratory effect of human Spry2 in HeLa cells is mediated by protein-tyrosine-phosphatase-1B (PTP1B) (13). More recent studies identified the receptor tyrosine kinase MET as an interaction partner through which human Spry2 positively regulates ERK activity and cell migration in response to hepatocyte growth factor signaling (18, 23). Interestingly, PTP1B as well as MET activity appear to be important for DC migration (79, 80). Taken together, these findings led us to speculate that Spry2 may be a promising candidate for regulating DC functions. We found that mouse and human Spry2 is expressed across multiple DC subsets at levels comparable to those in lymphocytes (**Figure 1A**; **Supplementary Figure S1B**). Interestingly, we found that Spry2 protein levels are negatively regulated in mouse BMDCs and human MoDCs upon maturation, indicating a regulatory role for Spry2 in DCs (**Figures 1C, D**; **Supplementary Figures S1C, D**). Nonetheless, we observed unaltered LPS-induced DC maturation in Spry2-deficient BMDCs (**Figures 1E, F**; **Supplementary Figures S2A, B**). In the study of Miyoshi and co-workers, mouse Spry2 overexpression in LM8 cells did not alter ERK activation in response to chemokine-induced CCR7 signaling. However, chemotaxis of LM8 cells towards the CCR7 ligands, CCL19 and CCL21, was significantly reduced, independently of ERK involvement (24). In line with this, we found that CCL19-induced ERK activation occurred with similar strength and kinetic in Spry2^+/+^ and Spry2^-/-^ mBMDCs (**Figures 4A, B**). Contrary to our expectations, however, the *in vitro* migration and *in vivo* LN homing efficiency of mBMDCs appeared to be unaffected by the loss of Spry2 (**Figures 3A–F**; **Supplementary Figures S3A, B**). Consistently, Spry2^-/-^ skin DCs showed unaltered emigration from skin explants and untaintedly homed to dLN in a FITC skin painting assay (**Figures 3G–M**). In addition, when we analyzed CD8^+^ T-cell priming *in vitro* as well as CD8^+^ T-cell effector differentiation during acute viral infection *in vivo*, DC priming capacity appeared unchanged by the lack of Spry2 (**Figures 4D–G**, **5A-I**). Of note, while ERK phosphorylation was unaffected by Spry2 deficiency, we observed a similar kinetic but marginally elevated levels of pAKT in Spry2^-/-^ mBMDCs at the peak 2 min after CCL19 stimulation (**Figures 4A, B**). AKT signaling has been linked to CCR7-dependent survival of mDCs (81), however, our additional results do not support any functional effect arising from the elevated pAKT levels observed in Spry2^-/-^ mDCs following CCR7 activation. Altogether, our findings indicate that Spry2 deficiency does not alter DC immune functions. In contrast, Spry2 deficiency was reported to significantly impact B- and T-cell responses with divergent effects (27–31). One notable difference is the regulation of Spry2 expression. In BMDCs, we observed a downregulation of Spry2 levels upon LPS-induced maturation. In contrast, Spry2 was generally reported to be upregulated following antigen receptor stimulation in B cells, T cells and in context of growth factor signaling (27, 31, 82–85). Moreover, an effect on enhanced memory formation was reported in Spry1/2 double KO CD8^+^ T cells, eliminating compensatory effects between Spry1 and 2, which share structural and functional features (30). In contrast, functional redundancy of Spry1 and Spry2 was ruled out in B cells, as Spry1 was undetectable with and without BCR stimulation (27). Whether compensatory mechanisms by other Spry proteins account for the unaltered activity of DCs in the absence of Spry2 remains to be elucidated. In summary, our findings demonstrate that Spry2-deficient DCs are functionally intact, exhibiting normal differentiation, antigen uptake, CCR7-directed migration, LN homing and CD8^+^ T-cell priming. Hence, our data provide insight into the function of Spry2 in DCs and contribute to a broader understanding of its diverse functions in immune cells.

## Data Availability

Datasets from this study are deposited on Zenodo and are publicly available under a Creative Commons Attribution 4.0 International license: 10.5281/zenodo.18848834.
