# Supplementary material for: Intact lymph node homing and CD8+ T-cell priming abilities of Sprouty2-deficient dendritic cells
Source: Front Immunol. 2026 Mar 18;17:1761675. doi: 10.3389/fimmu.2026.1761675 (PMC13038441; doi:10.3389/fimmu.2026.1761675)
Supplement: Supplementary file 1 [file DataSheet1.pdf]

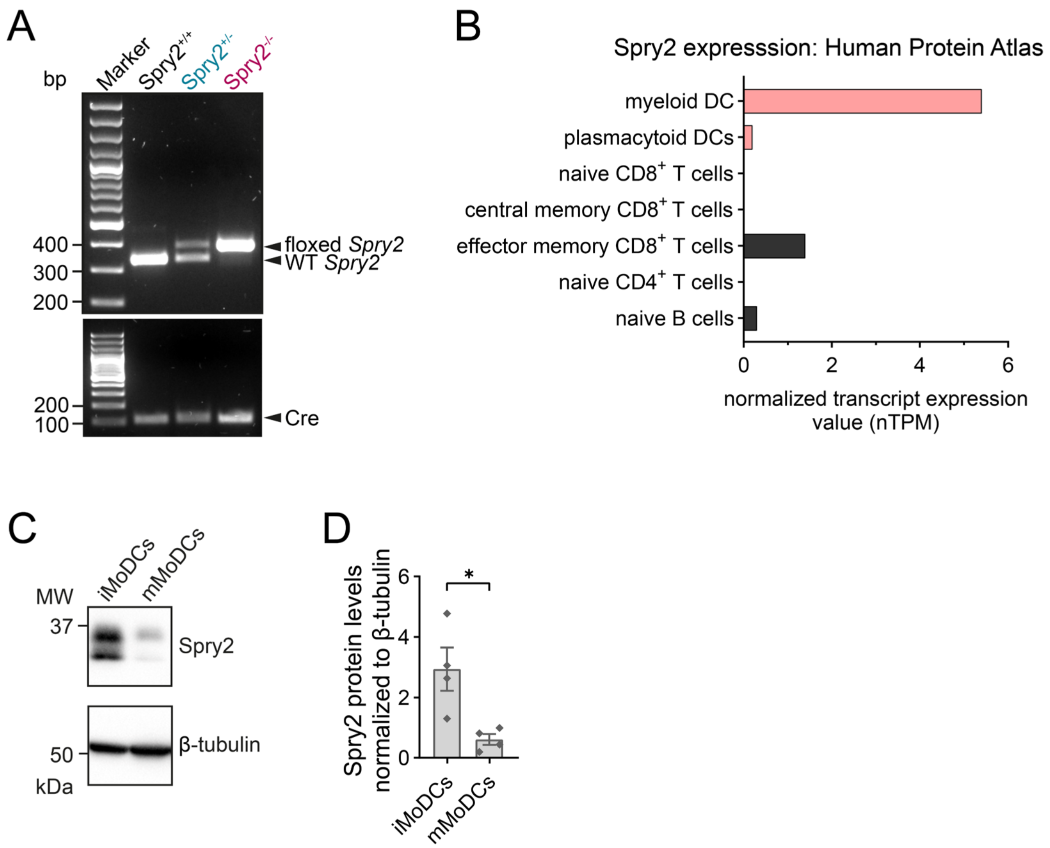

Supplementary Figure S1. Genotyping of mice for Spry2 deficiency and expression of Spry2 in human DCs. (A) Agarose gels of genomic PCR products from ear biopsies of *Spry2*$^{+/+}$*CD11c-Cre*$^{+}$ (Spry2$^{+/+}$), *Spry2*$^{+/fl}$*CD11c-Cre*$^{+}$ (Spry2$^{+/-}$) and *Spry2*$^{fl/fl}$*CD11c-Cre*$^{+}$ (Spry2$^{-/-}$) mice. PCR detection of loxP sites flanking the Spry2 ORF (upper panel) and the Cre transgene (lower panel). Spry2$^{+/+}$ mice carry two WT Spry2 alleles, Spry2$^{+/-}$ mice carry one WT and one floxed allele and Spry2$^{-/-}$ mice carry two floxed alleles. All genotypes express the Cre transgene. bp, base pairs. **(B)** Spry2 expression across human DC subsets and lymphocytes according to the Monaco dataset (62), retrieved from the Human Protein Atlas (accessed January 19, 2026). **(C-D)** Spry2 protein levels in immature (iMoDCs) and matured (mMoDCs) human monocyte-derived DCs (MoDCs) determined by Western blotting. **(C)** Representative Western blot showing Spry2 protein levels. **(D)** Quantification of Spry2 protein levels using densitometry. Bars show mean values ± SEM of MoDCs derived from 4 individual donors in 2 independent experiments. Statistical differences were determined using Student's unpaired t-test **(D)**; *$p \leq 0.05$.

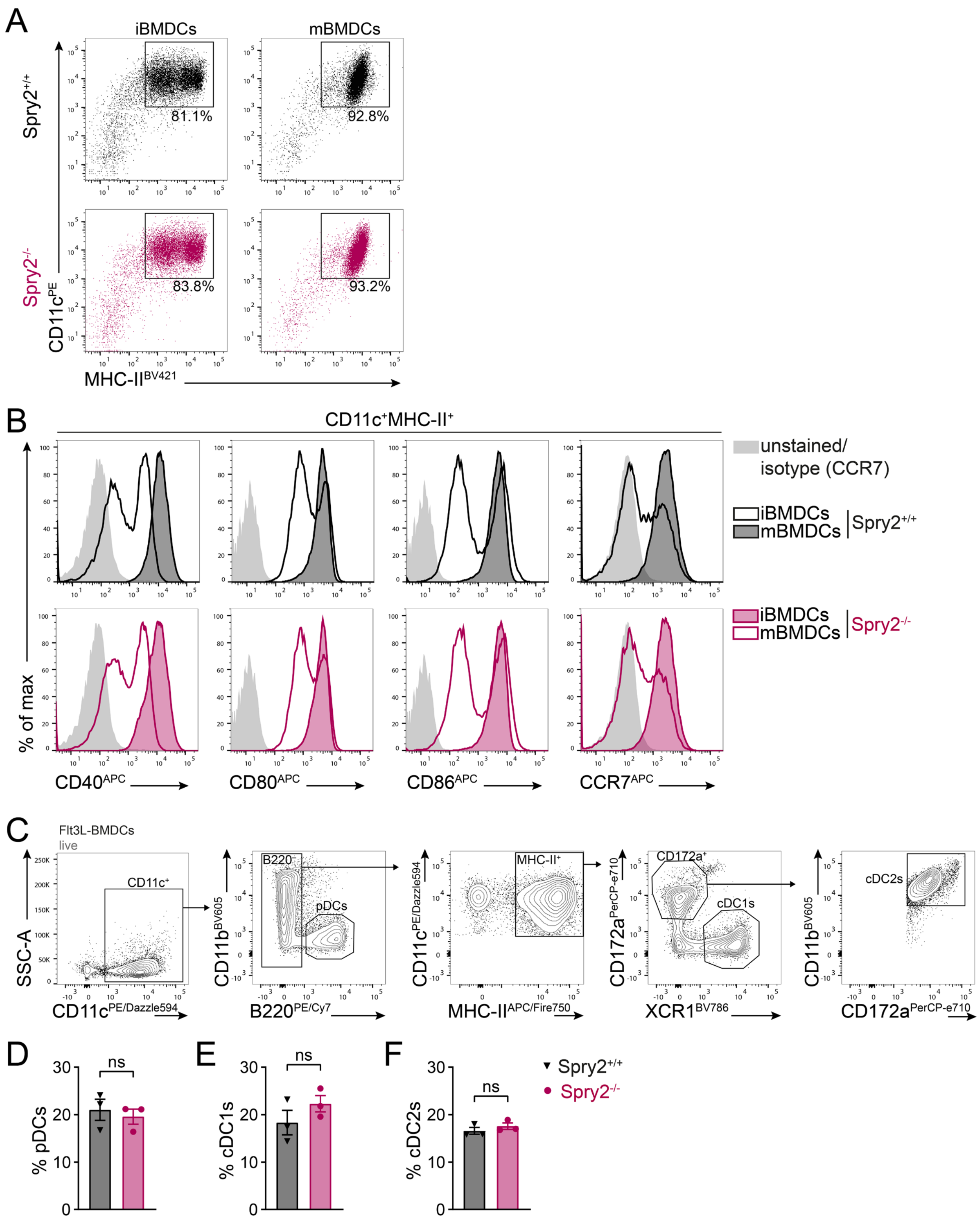

Supplementary Figure S2. Spry2-deficient BMDCs exhibit normal GM-CSF-mediated differentiation and maturation, and BM cells give rise to normal DC subsets in the presence of Flt3L. (A-B) BMDCs were generated by differentiating BM cells from $Spry2^{+/+}$ and $Spry2^{-/-}$ mice with GM-CSF for 8 days (iBMDCs), followed by LPS-induced maturation for 24 h (mBMDCs). Expression

of characteristic surface markers was assessed by flow cytometry. **(A)** Representative dot plots of CD11c^{+}MHC-II$^{int/high}$ iBMDCS and CD11c^{+}MHC-II^{high} mBMDCs (for quantification see Figure 1E). **(B)** Representative histograms of CD40, CD80, CD86 and CCR7 surface expression on CD11c^{+}MHC-II^{+} iBMDC and mBMDC populations (for quantification see Figure 1F). **(C-F)**. Flt3L-BMDCs were generated by differentiating BM cells from Spry2$^{+/+}$ and Spry2$^{-/-}$ mice with Flt3L for 8 days. Frequencies of plasmacytoid DCs (pDCs; CD11c^{+}CD11b^{-}B220^{+}), conventional DC1s (cDC1s; CD11c^{+}B220^{-}MHC-II^{+}XCR1^{+}) and cDC2s (CD11c^{+}B220^{-}MHC-II^{+}CD172a^{+}CD11b^{+}) present in the cultures were quantified by flow cytometry. **(C)** Gating strategy to determine DC subsets. **(D)** Frequencies of pDCs (% of live). **(E)** Frequencies of cDC1s (% of live). **(F)** Frequencies of cDC2s (% of live). Bars show mean ± SEM of Flt3L-BMDCs from 3 individual mice in 2 independent experiments. Statistical differences were determined using Student's unpaired t-test **(D, E, F)**; ns = not significant ($p > 0.05$).

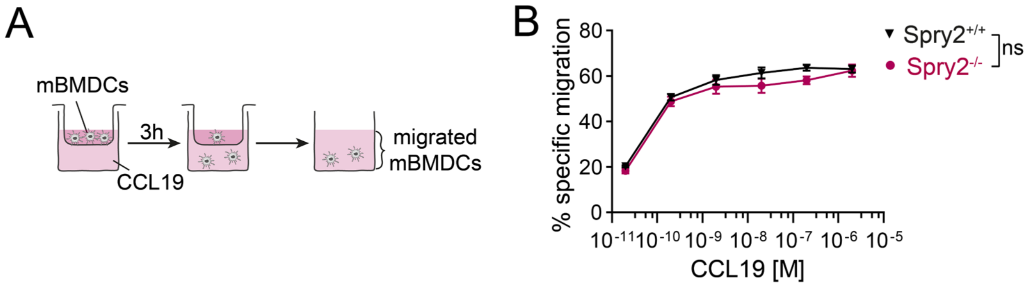

Supplementary Figure S3. Spry2-deficient DCs efficiently migrate towards graded concentrations of CCL19. (A-B) Spry2$^{+/+}$ and Spry2$^{-/-}$ mBMDCs were seeded in Transwell chemotaxis chambers and allowed to migrate towards graded concentrations of CCL19 (0.02 nM - 2 µM) for 3h. Migrated cells were quantified by flow cytometry. **(A)** Scheme of the 2D Transwell migration assay. **(B)** Quantification of specific migration. Mean values ± SEM of mBMDCs derived from 4 individual mice from 2 independent experiments. Statistical differences were determined using ordinary two-way ANOVA with Šídák's multiple comparisons test; ns = not significant ($p > 0.05$).
